# Canine Saliva as a Possible Source of Antimicrobial Resistance Genes

**DOI:** 10.3390/antibiotics11111490

**Published:** 2022-10-27

**Authors:** Adrienn Gréta Tóth, Imre Tóth, Bernadett Rózsa, Attila Dubecz, Árpád V. Patai, Tibor Németh, Selçuk Kaplan, Eszter Gabriella Kovács, László Makrai, Norbert Solymosi

**Affiliations:** 1 Centre for Bioinformatics, University of Veterinary Medicine, 1078 Budapest, Hungary; 2 Department of Operative Tecniques and Surgical Research, Faculty of Medicine, University of Debrecen, 4032 Debrecen, Hungary; 3 Department of Thoracic Surgery, Borsod-Abaúj-Zemplén County Hospital and University Teaching Hospital, 3526 Miskolc, Hungary; 4 Department of Small Animal Clinical Sciences, Western College of Veterinary Medicine, Saskatoon, SK S7N 5B4, Canada; 5 Department of Surgery, Paracelsus Medical University, 90419 Nuremberg, Germany; 6 Department of Surgery, Transplantation and Gastroenterology, Semmelweis University, 1085 Budapest, Hungary; 7 Interdisciplinary Gastroenterology Working Group, Semmelweis University, 1085 Budapest, Hungary; 8 Department and Clinic of Surgery and Ophthalmology, University of Veterinary Medicine, 1078 Budapest, Hungary; 9 Department of Agricultural Biotechnology, Tekirdag Namik Kemal University, Tekirdag 59030, Turkey; 10 Department of Microbiology and Infectious Diseases, University of Veterinary Medicine, 1143 Budapest, Hungary; 11 Department of Phyisics of Complex Systems, Eötvös Loránd University, 1117 Budapest, Hungary

**Keywords:** antimicrobial resistance, bacteriome, resistome, mobilome, dog saliva

## Abstract

While the One Health issues of intensive animal farming are commonly discussed, keeping companion animals is less associated with the interspecies headway of antimicrobial resistance. With the constant advance in veterinary standards, antibiotics are regularly applied in companion animal medicine. Due to the close coexistence of dogs and humans, dog bites and other casual encounters with dog saliva (e.g., licking the owner) are common. According to our metagenome study, based on 26 new generation sequencing canine saliva datasets from 2020 and 2021 reposited in NCBI SRA by The 10,000 Dog Genome Consortium and the Broad Institute within Darwin’s Ark project, canine saliva is rich in bacteria with predictably transferable antimicrobial resistance genes (ARGs). In the genome of potentially pathogenic *Bacteroides*, *Capnocytophaga*, *Corynebacterium*, *Fusobacterium*, *Pasteurella*, *Porphyromonas*, *Staphylococcus* and *Streptococcus* species, which are some of the most relevant bacteria in dog bite infections, ARGs against aminoglycosides, carbapenems, cephalosporins, glycylcyclines, lincosamides, macrolides, oxazolidinone, penams, phenicols, pleuromutilins, streptogramins, sulfonamides and tetracyclines could be identified. Several ARGs, including ones against amoxicillin–clavulanate, the most commonly applied antimicrobial agent for dog bites, were predicted to be potentially transferable based on their association with mobile genetic elements (e.g., plasmids, prophages and integrated mobile genetic elements). According to our findings, canine saliva may be a source of transfer for ARG-rich bacteria that can either colonize the human body or transport ARGs to the host bacteriota, and thus can be considered as a risk in the spread of antimicrobial resistance.

## 1. Introduction

Antimicrobial resistance (AMR) is a threat of utmost significance that constantly raises medical challenges all around the globe. It is a fact that the development of AMR and the increase in its prevalence are due to the use or misuse of antimicrobials. Moreover, according to the concept of One Health, the effects of antimicrobial use in human, animal and environmental sectors are interconnected, and thus interdependent. In the case of AMR, the relatedness of human and animal dimensions described by the One Health approach can be the best referred to by considering that most antimicrobial classes are co-used in both sectors. Human health antimicrobial use has been overshadowed for years by farm animal mass medication, although this tendency has recently changed in some parts of the world [1]. While the appearance and advance of AMR and, as an underlying cause, the enrichment and transmission of antimicrobial resistance genes (ARGs) in antibiotic-dense environments such as intensive animal production farms, is a well-examined phenomenon [2,3], the spread of AMR may also derive from other animal-borne routes.

Over the past decades, the number of companion animals has been tendentially and steadily rising [4]. Between 2000 and 2017, the number of dogs in the United States escalated from 68 million to 89.7 million [5]. In total, 67.9% of all households in the U.S. were associated with the ownership of various pet species and 48% of all with dogs in 2016 [4]. In the years 2019–2020, 50% of the U.S. population owned a dog [6]. The coronavirus disease (COVID-19) pandemic outbreak has resulted in elevated companion animal acquisition rates, albeit often followed by retention or replacement [7,8]. In addition to the popularity of keeping small animals, the quality of human–pet bonds has also changed. According to the survey of the American Veterinary Medical Association, 70% of pet owners consider their pets as family members, 17% as companions and 3% as property [9]. The role of pets can principally be defined as social companionship. Nowadays, having physical proximity is very common for pet–owner co-existences; pets often sleep together with their owners and lick their face or wounds [10]. Unfortunately and unsurprisingly, with such high dog numbers, the occurrence of dog bites is also common. Between 2001 and 2003, approximately 4.5 million dog bites were registered yearly in the United States, 19% of which necessitated medical intervention [11]. In the years 2005–2013, an average of 337,103 dog bite injuries were treated at U.S. emergency departments [12], although dog bites in general are under-reported [13]. In English hospitals, the number of dog bite admissions rose from 6.34 (95%CI 6.12–6.56) in 1998 to 14.99 (95%CI 14.67–15.31) admissions per 100,000 inhabitants in 2018 [14]. In parallel, a study on the database of The International Statistical Classification of Diseases and Related Health Problems, Tenth Revision (ICD-10) reveals that dog attack fatalities (excluding deaths caused by postattack infectious complications) have been constantly rising between 1995 and 2016. Incidence rates reached 0.009 per 100 000 inhabitants in Europe, 0.011 in the U.S., 0.007 in Canada and 0.004 in Australia [15]. Interestingly, 3 of 5 bites are executed by family dogs, which is more common than attacks by strays [16].

The modern mindset of providing regular veterinary healthcare services to our pets and keeping them in our closest surroundings may contribute to the interspecies transmission of AMR. Several studies have already turned their attention to the role of companion animals in the headway of AMR [17,18,19,20,21,22]. Nevertheless, the significance of the direct pet-borne AMR spread route has been given less attention when compared with the rather indirect, mostly food-transmitted farm-animal-associated route. The routes of ARG transmission can be assessed by analyzing the genetic surroundings of ARGs. Genetic elements that facilitate horizontal gene transfer (HGT) including plasmids, bacteriophages and integrative mobile genetic elements (iMGEs) may contribute to three different HGT processes, namely conjugation, transduction or transformation. By conjugation, genes are delivered to a recipient cell on plasmids if bacterial cells are physically binded, while by transduction, direct cell-to-cell contact is disclaimed due to the presence of bacteriophages as means of gene conduit. Transformation negates the need for particular delivery processes. In this case, bacteria take up genetic fragments from their environment [23]. After dog bites or close encounters with saliva from dogs that undergo veterinary treatments, and thus may carry bacteria with a possibly enriched ARG content, resistant bacteria may be introduced to the human body, and later the HGT of antimicrobial resistance determinants may be exchanged with the host bacteriota. Our study aimed to reveal the ARG content of canine saliva samples, attach the ARGs with the bacterial species that they derive from and report the ARGs’ spreading capabilities to weigh the above-mentioned phenomenon. For this purpose, freely accessible next-generation sequencing (NGS) shotgun datasets were downloaded and bioinformatically analyzed using an advanced metagenomic pipeline.

## 2. Materials and Methods

Deep-sequenced canine saliva datasets were searched in the National Center for Biotechnology Information (NCBI) Sequence Read Archive (SRA) repository. In May 2021, two shotgun metagenomic BioProjects (PRJNA648123 [24]—The 10,000 Dog Genome Consortium and PRJNA683923 [25]—Broad Institute, Darwin’s Ark project) with more than 100,000,000 paired-end reads per sample were identified (Table 1). Both projects collected and sequenced dog saliva samples to investigate polymorphisms in the dog genome from which the samples were derived. The median read count (interquartile range, IQR) of the samples was $177.7\times 10^{6}$ ($26.6\times 10^{6}$) and $417.7\times 10^{6}$ ($90.1\times 10^{6}$) in datasets PRJNA648123 and PRJNA683923, respectively.

### Bioinformatic Analysis

Quality-based filtering and trimming of the raw short reads was performed by TrimGalore (v.0.6.6, https://github.com/FelixKrueger/TrimGalore, accessed on 24 September 2022), setting 20 as a quality threshold. Only reads longer than 50 bp were retained and taxonomically classified using Kraken2 (v2.1.1) [26] and a database created (24 March 2021) from the NCBI RefSeq complete archaeal, bacterial, viral and plant genomes. For this taxon assignment, the–confidence 0.5 parameter was used to obtain more precise species-level hits. The taxon classification data were managed in R [27] using functions of the packages phyloseq (v1.36.0) [28] and microbiome (v1.14.0) [29]. Reads classified as origin of bacteria were assembled to contigs by MEGAHIT (v1.2.9) [30] using default settings. The contigs were also classified taxonomically by Kraken2 with the same database as above. All possible open reading frames (ORFs) were gathered by Prodigal (v2.6.3) [31] from the contigs. The protein-translated ORFs were aligned to the ARG sequences of the Comprehensive Antibiotic Resistance Database (CARD, v.3.1.3) [32,33] by Resistance Gene Identifier (RGI, v5.2.0) with Diamond [34]. The ORFs classified as perfect or strict were further filtered with 90% identity and 90% coverage. All nudged hits were excluded. The iMGE content of the ARG-harboring contigs was analyzed by MobileElementFinder (v1.0.3) and its database (v1.0.2) [35]. Following the distance concept of Johansson et al. [35] for each bacterial species, only those with a distance threshold defined within iMGEs and ARGs were considered associated. In the MobileElementFinder database (v1.0.2) for *Bacteroides*, the longest composite transposon (cTn) was the *Tn6186*. In the case of this genus, its length (8505 bp) was taken as the cut-off value. For the genera *Enterococcus* and *Klebsiella*, *Tn6246* (5147 bp) and *Tn125* (10,098 bp) provided the thresholds, respectively. In the case of *Escherichia coli*, this limit was the length of the *Tn1681* transposon, namely 24,488 bp, while for *Pseudomonas aeruginosa Tn6060* (25,440 bp). As the database neither contains species-level, nor genus-level cTn data for the rest of the species, a general cut-off value was chosen for the contigs of these species. This value was declared as the median of the longest cTns per species in the database (10,098 bp). The plasmid origin probability of the contigs was estimated by PlasFlow (v.1.1) [36]. The prophage content of the assembled contigs was prediced by VirSorter2 (v2.2.3) [37]. The findings were filtered for dsDNAphages and ssDNAs. All data management procedures, analyses and plottings were performed in R environment (v4.1.0) [27].

## 3. Results

After presenting the bacteriome and the identified AGRs (resistome), predictions regarding the mobility potential of ARGs were also resumed based on genetic characteristics that may play a significant role in HGT.

### 3.1. Bacteriome

**Figure 1:**
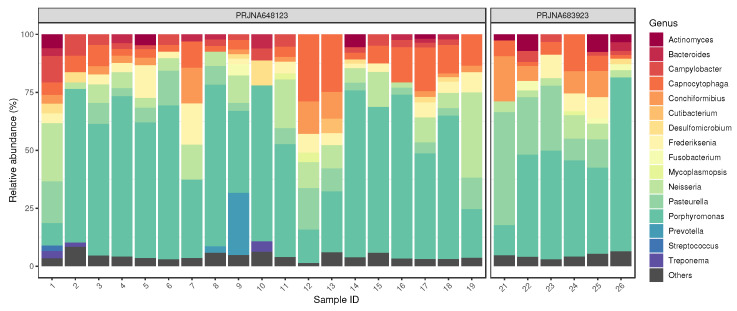
Saliva core bacteriome. The relative abundances of genera that achieved more than 1% of the bacterial hits in any of the samples. In the sample No. 20, no reads were classified to bacteria.

By taxon classification, the number of reads aligning to bacterial genomes differed in the various samples. In the saliva, median bacterial read count of the samples was $4.3\times 10^{6}$ (IQR: $3.4\times 10^{6}$). A total of 16 major bacterial genera were detected within the saliva samples, out of which several aerobic and anaerobic genera often become isolated from infected dog bite wounds. The relative abundances of genera that achieved more than 1% of the bacterial hits in any of the saliva samples is shown in Figure 1. In the saliva samples, the dominant genera (with mean prevalence) in descending order were *Porphyromonas* (49%), *Prevotella* (15%), *Pasteurella* (12%), *Neisseria* (10%), *Capnocytophaga* (9%), *Conchiformibius* (7%), *Frederiksenia* (7%), *Cutibacterium* (6%), *Actinomyces* (5%), *Campylobacter* (4%), *Desulfomicrobium* (4%), *Bacteroides* (3%), *Fusobacterium* (3%), *Mycoplasmopsis* (3%), *Treponema* (3%) and *Streptococcus* (2%). In the sample No. 20, no reads were classified to bacteria.

### 3.2. Resistome

**Figure 2:**
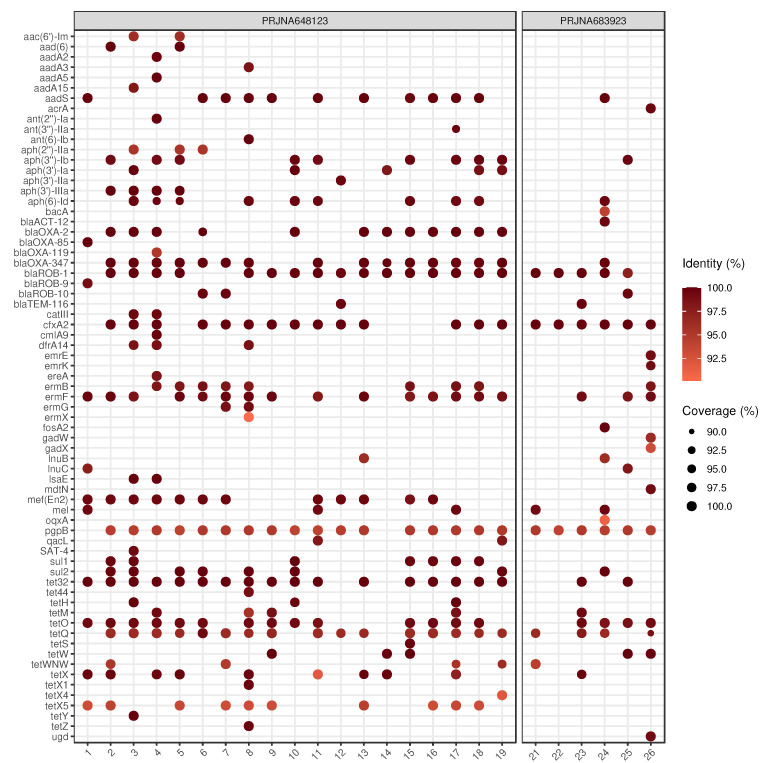
Identifed ARGs by samples. For each sample–ARG combination, only the best finding is plotted. The size and the color of the dots correspond to the coverage and the sequence identity of hits on reference genes, respectively. In sample No. 20, there was no identifiable ARG. The gene names that are too long are abbreviated (*acrA*: *E. coli acrA*; *emrE*: *E. coli emrE*).

Applying the above-mentioned filtering conditions, we identified 69 ARGs that are presented together with their prevalence rates within the samples and the drug classes that these ARGs affect adversely in Table 2. These ARGs per sample with their coverage and the sequence identity rate are shown in Figure 2. As a result of the taxon classification on the contigs harboring the ARGs, it was possible to predict the bacterial species of putative origin for all but ten genes (Table 3). The dominant mechanisms of identified ARGs were antibiotic inactivation (47.69%), antibiotic target protection (23.41%), antibiotic target alteration (15.90%), antibiotic efflux (7.80%) and antibiotic target replacement (5.20%).

### 3.3. Mobilome

**Figure 3:**
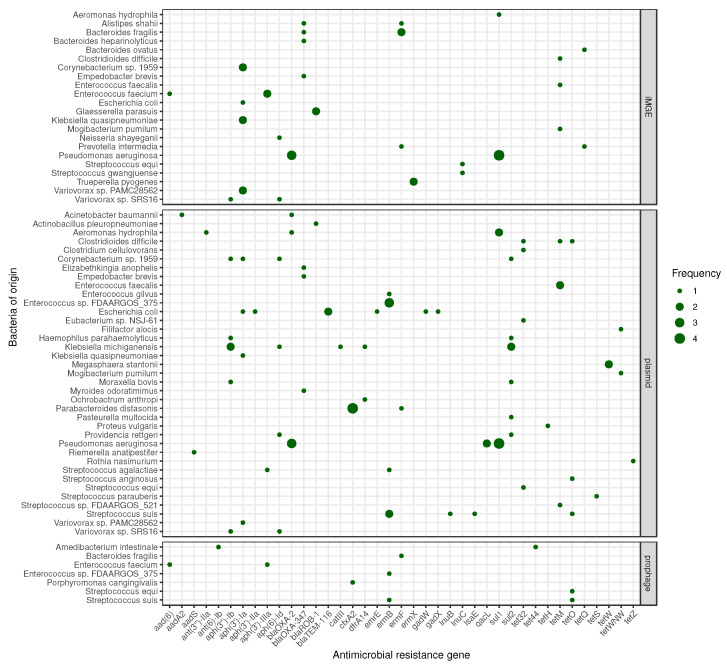
Mobile ARG frequency by bacteria of origin. The size of the dots indicates the occurrence frequency of the given gene flanked by iMGE, positioned in plasmid or prophage.

Many of the identified ARGs are harbored by iMGEs, prophages or plasmids. The frequencies of iMGEs, prophages and plasmids associated with ARGs by bacteria of origin are summarized in Figure 3. Some genes could have been attached to two of the above-mentioned mobility groups in the genome of one species, including the iMGE and prophage co-appearance of aminoglycoside resistance encoding *aad(6)* and *aph(3’)-IIIa* in *E. faecium*; the iMGE and plasmid co-appearance of *aph(3’)-Ia* in *Corynebacterium* sp. 1959 and *K. quasipneumoniae*; *aph(3’)-Ia* and *aph(6)-Id* in *Variovorax* sp. SRS16; *aph(3”)-Ib* in *Variovorax* sp. PAMC28562; tetracycline resistance encoding *tetM* in *E. faecalis*; and prophage and plasmid co-appearance of macrolide, lincosamide and streptogramin resistance encoding *ermB* in *Enterococcus* sp. FDAARGOS_375. The *blaOXA-2*, *blaOXA-347* and *blaTEM-116* genes associated with amoxicillin–clavulanate resistance all appeared in plasmids in various species; moreover, *blaOXA-2* was associated with both an iMGE and a plasmid in the genome of *P. aeruginosa*.

## 4. Discussion

During the bacteriome, resistome and mobilome analysis of the canine saliva samples, a large set of results was obtained that can be examined from a One Health point of view, merging the small animal veterinary sector with the perspective of the human healthcare system.

ARGs were identified in all but one sample (No. 20). No reads of bacterial origin were found in this sample. We speculate that this may be since only those reads generated from sequencing were uploaded to the SRA mapped to the dog genome.

Many of the detected aerobic and anaerobic bacterial genera often become isolated from infected dog bite wounds. Dog bite infections are normally polymicrobial, and the bite wound bacteriota consist of bacteria from the animals’ oral cavity, the recipients’ skin and the environment. The most common pathogens in dog bites are *Pasteurella* spp. (*P. canis* and *P. multocida*), *Staphylococcus* spp., *Streptococcus* spp. and *Capnocytophaga* spp., *Porphyromonas* spp., *Bacteroides* spp., *Fusobacterium* spp. and *Corynebacterium* spp. [38], which all appeared in the analyzed saliva samples. Some other bacterial groups of a relatively higher clinical significance that were detected in the saliva samples, including *Enterococcus* spp., *Moraxella* spp., *Neisseria* spp., *Prevotella* spp. and *Pseudomonas* spp., are also often isolated from dog bite wound infections. The vast majority of other genera isolated in the samples have been mentioned to appear in dog saliva in previous publications with variable abundance rates [13,39]. Even though membersof *Clostridium* spp. were detected in the samples, genome fragments of *C. tetani*, the bacterium responsible for tetanus, were not identified. The number of detected ARGs was relatively high in the salivary bacteriome. Examining eight genera (*Pasteurella* spp., *Staphylococcus* spp., *Streptococcus* spp., *Capnocytophaga* spp., *Porphyromonas* spp., *Bacteroides* spp., *Fusobacterium* spp. and *Corynebacterium* spp.) that were indicated to be the most relevant ones in dog bite infections by other authors [38,39], we could identify genes that confer resistance against aminoglycosides, carbapenems, cephalosporins, glycylcyclines, lincosamides, macrolides, oxazolidinone, penams, phenicols, pleuromutilins, streptogramins, sulfonamides and tetracyclines, while other antimicrobial groups including fluoroquinolones appeared in *E. coli*, one of the six leading pathogens responsible for the deaths associated with resistance in 2019 worldwide [40].

Such a great number and broad spectrum of ARGs and potentially affected antimicrobial groups associated with the canine saliva samples may be related to the use of antibiotics at small-animal veterinary practices. Antibiotic consumption rates in the companion animal sector are rather difficult to evaluate. However, some systems exist for the surveillance of magnitude of companion animal antibiotic consumption, such as the European Surveillance of Veterinary Antimicrobial Consumption (ESVAC) [41], VetCompass [42] or the Small Animal Veterinary Surveillance Network (SAVSNET) [43], these rates are still less well-documented. Moreover, in many countries, antimicrobial use is often just estimated by rough sales data [21]. Nevertheless, according to the two UK-based surveillance systems (VetCompass from Royal Veterinary College, and SAVSNET from Liverpool University) and one EU report (ESVAC), antibiotics are rather frequently prescribed at small-animal clinics. A study states 1 in 4 UK dogs (25.2%, 95% CI: 25.1–25.3%) were treated with antibiotics in a two-year period [44]. Even though the vast majority of veterinarians are aware of the fact that improper AMU contributes to selection for AMR, and that it is a significant problem according to nationwide surveys [45,46], there are many factors that influence the antibiotic prescription preferences of veterinarians in addition to the perspectives of antimicrobial stewardship [47,48,49,50,51,52].

Broad-spectrum amoxicillin–clavulanate is the flagship of antimicrobial agents applied in dogs in many countries, while first-generation cephalosporins are also routinely used [21,53,54]. Lincosamides (clindamycin), macrolides, tetracyclines (doxycycline), nitroimidazoles and trimethoprim/sulphonamides have also been reported to be frequently used in small-animal practices [21]. Third- and fourth-generation cephalosoprines, fluoroquinolones and polymixins that belong to cathegory B, ‘last resort’, or highest-priority Critically Important Antibiotics (HPCIAs) according to the European Medicines Agency [41] should be avoided unless sensitivity testing is conducted and no other antibiotics would be effective. Nevertheless, HPCIAs have been estimated to be prescribed in around 5–6% of total antimicrobial agent usage events. Of the HPCIA category, fluoroquinolones are the most common in dogs, constituting ∼4 to 5% of total antibiotic prescriptions [55].

Neverheless, AMR determinants against the above-mentioned antimicrobial compound groups have been detected in and associated with many bacterial species of the examined canine saliva samples. Some of these ARG-associated bacteria can also exert pathogenic effects and are often isolated by dog bite infections. ARGs against cephalosporins were identified in many, often clinically significant bacteria, including but not limited to *Bacteroides* spp., *Capnocytophaga* spp., *E. coli*, *F. ulcerans*, *Porphyromonas* spp. and *P. aeruginosa*. Likewise, ARGs against lincosamides appeared in *Bacteroides* spp., *C. stomatis*, *Enterococcus* spp., *P. cangingivalis*, *P. intermedia* and *Streptococcus* spp.; ARGs against macrolides in *Bacteroides* spp., *C. stomatis*, *Enterococcus* spp., *Porphyromonas* spp., *P. intermedia* and *Streptococcus* spp.; ARGs against tetracyclines in *Bacteroides* spp., *C. stomatis*, *Enterococcus* spp., *P. crevioricanis*, *Prevotella* spp., *P. putida* and *Streptococcus* spp.; ARGs against sulfonamides in *Corynebacterium* spp., *M. bovis*, *P. multocida* and *P. aeruginosa*; and ARGs against fluoroquinolones in *E. coli* (non-exhaustive lists of detected ARG-bacteria associations in the samples, with an emphasis on clinically relevant bacteria). Bacterial associations and the significance of AMR determinants affecting amoxicillin–clavulanate, the most commonly prescribed antibiotic in veterinary medicine, are discussed later on. Even though nitroimidazoles (e.g., metronidazole) are described to be often used in small-animal veterinary practices, no ARGs could be detected in the canine saliva samples against this antibiotic group.

In the current literature, human infections associated to dog bites are better and more frequently documented than the transmission route of licking. Three to thirty percent of dog bites lead to infection [13]. The management of animal bites rests on two pillars: local wound care and adequately applied systemic treatment. Essentials of local therapy include inspection, debridement of the wound accompanied by the removal of possible foreign bodies, e.g., teeth, and irrigation with saline solution. As for the systematic therapy, tetanus boosters (if none given in the past year) and rabies prophylaxis should always be considered. In our study, genome fragments of *C. tetani*, the causative agent of tetanus, were not detected in any of the examined saliva samples. No consensus has yet been found in the use of antibiotics for animal bite wound care. Prophylactic antibiotics should be considered unless the wound is very superficial and clean. Explicit indications for antibiotic prophylaxis or therapy include presentation at least 8 h after the bite, clear signs of superinfection, moderate or severe wounds with crush injuries or devitalized tissues requiring surgery, deep puncture wounds (exceeding the layer of epidermis), wounds close to joints, diabetes mellitus, asplenic or immunocompromised state, alcohol abuse, or involvement of the genital area, face or hand [56,57,58,59,60]. In the absence of the above reasons, antibiotic therapy may not be necessary. Interestingly, injuries are normally located on the head, neck and face in children and on the hand or upper extremity in adults due to height ratios with the attacking dog [13,61]. An adequately chosen antibiotic agent is expected to be effective against anaerobe bacteria (*Bacteroides* spp., *Fusobacterium* spp., *Porphyromonas* spp., *Prevotella* spp. etc.), in addition to the *Staphylococcus*, *Streptococcus* and *Pasterurella* species. Prophylactic treatment is normally 3 to 5 days long, while medication for 10 days or longer is recommended if the wound is infected. The first-line choice for oral therapy is amoxicilin–clavulanate, accompanied with a first dose of intravenous antibiotic (e.g., ampicillin/sulbactam, ticarcillin–clavulanate, piperacillin–tazobactam, or a carbapenem) in high-risk patients. Amoxicillin–clavunalate is often combined with metronidazole or clindamycin and is also sometimes replaced with cephalosporins, e.g., cefuroxime, cefotaxime, ceftriaxone or amoxicillin, fluoroquinolones, sulfamethoxazole and trimethoprim, and alternatively, although less effective, azithromycin or doxycycline in this combination [58,60]. Due to high resistance rates, flucloxacillin, erythromycin and cephalosporins are often ineffective in *Pasteurella* infections, and thus should rather be avoided [57]. In our case, no genes conferring resistance to these agent groups could be identified in *Pasturella* spp.

Data on the outcome of antibiotic prophylaxis in animal bite management by humans is limited and rather controversial and conflicting. While a meta-analysis of eight randomized trials indicated a benefit of antibiotic prophylaxis [62], some studies concluded that antibiotic prophylaxis does not result in a statistically significant difference in the frequency of wound infections among treated and untreated patient groups, except for wounds to the hand [63]. Based on other publications, antibiotic prophylaxis should be recommended for high-risk patient groups only [64,65].

Based on antibiotic prescription data from human and veterinary medical practices described above, amoxicillin–clavulanate and cephalosporins are the most commonly used agents in the treatment of animal patients and dog bite infections, while lincosamides (mostly clindamycin), sulfonamides (mostly potentiated sulfonamides) and fluoroquinolones also appear in both sectors [21,53,54,55,57,58,61].

Amoxicillin–clavulanate, the most commonly used antibiotic in small-animal medicine, and the first choice for canine bite wounds, is a member of broad-spectrum penicillins that have been a frequently consumed key antibiotic group in the high-income super-region between 2000 and 2018 according to a global study [66]. All in all, six ARG types were detected in the dog saliva samples that may confer resistance against amoxicillin–clavulanate, which were either the members of the *blaTEM* or *OXA* family [67,68]. *blaTEM-116* was identified in *E. coli*, while various members of the *OXA* family appeared in in many genera, including *A. baumannii*, *Bacteroides* spp., *Capnocytophaga* spp., *F. ulcerans* and *Pseudomonas* spp., which can have a high clinical relevance in dog bite infections. Moreover, plasmid-borne *blaOXA-2*, *blaOXA-347* and *blaTEM-116* genes in different bacteria, and *OXA-2* associated with both an iMGE and a plasmid from *P. aeruginosa*, all confer resistance against amoxicillin–clavulanate and have a higher potential to spread from bacteria to bacteria. The accumulation of various mobility factors around the genes may increase the chance of the horizontal transfer of the given ARG. The canine saliva-borne transmission of bacteria harboring mobile ARGs may hamper antimicrobial use in human clinical settings and can also contribute to the spread of AMR among the bacteria derived from pets to the bacteriota appearing in humans.

Cephalosporins, which are also commonly used both in companion animals medicine [21,54] and human medicine, including in protocols for dog bite infections [58,61], have been associated with all in all twenty-four bacterial species harboring related ARGs in the canine saliva samples. These bacteria include pathogens such as *A. baumanni*, *Bacteroides* spp., *Capnocytophaga* spp., *F. ulcerans* and *P. aeruginosa* that contain *blaOXA-2*, *blaOXA-347*, *blaOXA-347*, *blaOXA-85* and *blaOXA-2*, respectively. Importantly, *P. aeruginosa*-associated *blaOXA-2*, which was associated with both an iMGE and a plasmid, has a high potential for HGT and can be considered as a major public health concern.

Lincosamides, including clindamycin, are also significant in both veterinary medicine [21,54] and the treatment of dog bite cases in humans [58,61]. Genes affecting lincosamides appeared in seventeen bacterial species, many of which, e.g., *B. fragilis* (*ErmF*), *C. stomatis* (*ErmF*), *Porphyromonas* spp. (*ErmF*) and *Streptococcus* spp. (*ErmB*, *lnuB* and *lnuC*), are potentially pathogenic. While the above-mentioned species, which are often associated with dog bite infections, contained no genetic element around these ARGs that could facilitate their transfer, an *Enterococcus* species from the canine saliva samples is linked to carrying *ermB* with prophage and plasmid co-appearance. Thus, the possibility of the transfer of this gene to other bacteria with higher clinical significance is given in the case of lincosamides as well.

Sulfonamide resistance genes appeared in relatively fewer, namely eight, bacterial species, including *P. multocida* and *P. aeruginosa* harboring *sul2* and *sul1*, respectively. In addition to the low ARG counts, none of the sulfonamide resistance genes appeared to be mobile, while, in contrast, HGT has been found to be highly characteristic for these two genes in other publications [69].

In the case of fluoroquinolones, only *E. coli* harbored related ARGs, namely the non-mobile *acrA*, *gadW* and *gadX*. Interestingly, while both *gadW* and *gadX* are *AraC* family regulators that promote *mdtEF* expression to confer multidrug resistance, when they co-occur, *gadW* inhibits *gadX*-dependent activation by repressing *gadX* (CARD).

Furthermore, fosfomycin and tygecyclin, which are often used as last-resort antimicrobial agents [70,71] and are involved in the list of Critically Important Antibiotics for Human Medicine by WHO [72], also appeared among the affected antibiotic groups, namely due to the presence of *fosA2* and *tetX4*, *tetX5*, respectively. Nonetheless, while the members of the *tet(X)* family are often plasmid-associated [73,74,75,76], their plasmid relatedness was not predicted within this study.

While the above-mentioned findings may raise awareness of the potential public health risk associated with canine saliva, this material has been used to promote rapid healing and to reduce bacterial contamination in the past according to the reports of ethnoveterinary and ethnomedicinal practices [77,78]. Antimicrobial and anti-imflammatory activity of canine saliva induced by thiocyanate, lysozyme and, indirectly, nitrate, among others [79,80], can even appear at low concentrations [81]. However, according to our findings, canine saliva can also be associated with public health risks, since salival bacteria may contaminate the surroundings of people and may also colonize human skin and mucous membranes. Thus, ARG-rich bacteria present in and around humans do not even necessarily need to transfer their ARGs to potentially cause severe harm to various groups of people with weaknesses of the immune system, e.g., extremities in age or diseased state.

As a common trend among many nations, veterinary use of antibiotics is gradually declining [49,55,81,82,83]. In human medicine, antibiotic sales elevated by 65% in low- and middle-income countries and decreased slightly by 4% in high-income countries between 2000 and 2015, which adds up to a rise in global antibiotic consumption rates [66,84].As a presumable conclusion, several genes conferring resistance against clinically important antimicrobial groups are present in the salivary bacteriome of dogs that may drift to the genome of bacteria in humans. Encounters with dog saliva and dog bites may serve as an interspecies platform for the migration of bacteria and ARGs. Transmitted bacteria may cause clinical symptoms, and ARGs that they harbor may confer resistance against antimicrobial agents of a clinical relevance.

## Figures and Tables

**Table 1:** The list of analyzed samples was obtained from the National Center for Biotechnology Information Sequence Read Archive. Column Run contains the NCBI SRA run identifiers. Bacterial read count represents the number of reads that were classified taxonomically to any bacteria.

ID	BioProject	Run	Bacterial Read Count
1	PRJNA648123	SRR12330029	2,900,387
2		SRR12330041	16,153,172
3		SRR12330042	13,072,781
4		SRR12330043	13,774,332
5		SRR12330044	6,123,646
6		SRR12330045	16,707,766
7		SRR12330098	18,826,266
8		SRR12330104	27,598,592
9		SRR12330220	9,938,948
10		SRR12330260	17,642,933
11		SRR12330298	17,277,697
12		SRR12330356	13,988,719
13		SRR12330364	17,378,513
14		SRR12330377	12,155,726
15		SRR12330378	34,183,357
16		SRR12330382	22,353,314
17		SRR12330383	22,886,951
18		SRR12330384	18,328,656
19		SRR12330385	6,631,504
20	PRJNA683923	SRR13340534	0
21		SRR13340535	6,752,169
22		SRR13340537	8,245,374
23		SRR13340538	41,212,470
24		SRR13340539	13,028,655
25		SRR13340540	6,964,460
26		SRR13340541	6,279,921

**Table 2:** Identified ARGs and the drug classes affected by them. The frequency columns show how many samples the genes occurred in.

ARG(s)	Frequency		Drug Class
	n	%	
*aac(6’)-Im*	2	7.7	aminoglycoside
*aad(6)*	2	7.7	aminoglycoside
*aadA2*	1	3.8	aminoglycoside
*aadA3*	1	3.8	aminoglycoside
*aadA5*	1	3.8	aminoglycoside
*aadA15*	1	3.8	aminoglycoside
*aadS*	12	46.2	aminoglycoside
*acrA*	1	3.8	cephalosporin, fluoroquinolone, glycylcycline, penam, phenicol, rifamycin, tetracycline, triclosan
*ant(2”)-Ia*	1	3.8	aminoglycoside
*ant(3”)-IIa*	1	3.8	aminoglycoside
*ant(6)-Ib*	1	3.8	aminoglycoside
*aph(2”)-IIa*	3	11.5	aminoglycoside
*aph(3”)-Ib*	10	38.5	aminoglycoside
*aph(3’)-Ia*	5	19.2	aminoglycoside
*aph(3’)-IIa*	1	3.8	aminoglycoside
*aph(3’)-IIIa*	4	15.4	aminoglycoside
*aph(6)-Id*	10	38.5	aminoglycoside
*bacA*	1	3.8	peptide
*blaACT-12*	1	3.8	carbapenem, cephalosporin, cephamycin, penam
*blaOXA-2*	12	46.2	carbapenem, cephalosporin, penam
*blaOXA-85*	1	3.8	carbapenem, cephalosporin, penam
*blaOXA-119*	1	3.8	carbapenem, cephalosporin, penam
*blaOXA-347*	16	61.5	carbapenem, cephalosporin, penam
*blaROB-1*	21	80.8	cephalosporin, penam
*blaROB-9*	1	3.8	cephalosporin, penam
*blaROB-10*	3	11.5	cephalosporin, penam
*blaTEM-116*	2	7.7	cephalosporin, monobactam, penam, penem
*catIII*	2	7.7	phenicol
*cfxA2*	20	76.9	cephamycin
*cmlA9*	1	3.8	phenicol
*dfrA14*	3	11.5	diaminopyrimidine
*emrE*	1	3.8	macrolide
*emrK*	1	3.8	tetracycline
*ereA*	1	3.8	macrolide
*ermB*	9	34.6	lincosamide, macrolide, streptogramin
*ermF*	18	69.2	lincosamide, macrolide, streptogramin
*ermG*	2	7.7	lincosamide, macrolide, streptogramin
*ermX*	1	3.8	lincosamide, macrolide, streptogramin
*fosA2*	1	3.8	fosfomycin
*gadW*	1	3.8	fluoroquinolone, macrolide, penam
*gadX*	1	3.8	fluoroquinolone, macrolide, penam
*lnuB*	2	7.7	lincosamide
*lnuC*	2	7.7	lincosamide
*lsaE*	2	7.7	lincosamide, macrolide, oxazolidinone, phenicol, pleuromutilin, streptogramin, tetracycline
*mdtN*	1	3.8	acridine dye, disinfecting agents and intercalating dyes, nucleoside
*mef(En2)*	12	46.2	macrolide
*mel*	5	19.2	lincosamide, macrolide, oxazolidinone, phenicol, pleuromutilin, streptogramin, tetracycline
*oqxA*	1	3.8	diaminopyrimidine, fluoroquinolone, glycylcycline, nitrofuran, tetracycline
*pgpB*	23	88.5	peptide
*qacL*	2	7.7	disinfecting agents and intercalating dyes
*SAT-4*	1	3.8	nucleoside
*sul1*	7	26.9	sulfonamide
*sul2*	8	30.8	sulfonamide
*tet32*	19	73.1	tetracycline
*tet44*	1	3.8	tetracycline
*tetH*	3	11.5	tetracycline
*tetM*	5	19.2	tetracycline
*tetO*	18	69.2	tetracycline
*tetQ*	20	76.9	tetracycline
*tetS*	1	3.8	tetracycline
*tetW*	5	19.2	tetracycline
*tetWNW*	5	19.2	tetracycline
*tetX*	10	38.5	glycylcycline, tetracycline
*tetX1*	1	3.8	tetracycline
*tetX4*	1	3.8	glycylcycline, tetracycline
*tetX5*	10	38.5	tetracycline
*tetY*	1	3.8	tetracycline
*tetZ*	1	3.8	tetracycline
*ugd*	1	3.8	peptide

**Table 3:** Identified ARGs and the predicted bacterial species of origin. For ten genes (*aadA5*, *aadA15*, *ant(2”)-Ia*, *bacA*, *blaACT-12*, *cmlA9*, *fosA2*, *oqxA*, *tetX1* and *tetY*), no species-level prediction was obtained as to which bacterium the contig carrying the gene might have originated from.

ARG(s)	Bacteria of Origin
*aac(6’)-Im*	*Clostridioides difficile*
*aad(6)*	*Enterococcus faecium*, *Staphylococcus aureus*
*aadA2*	*Acinetobacter baumannii*
*aadA3*	*Neisseria animaloris*
*aadS*	*Bacteroides fragilis*, *Capnocytophaga* sp. H2931, *Capnocytophaga* sp. H4358, *Capnocytophaga stomatis*, *Chryseobacterium indologenes*, *Riemerella anatipestifer*
*acrA*	*E. coli*
*ant(3”)-IIa*	*Aeromonas hydrophila*
*ant(6)-Ib*	*Amedibacterium intestinale*
*aph(2”)-IIa*	*C. difficile*
*aph(3”)-Ib*	*Corynebacterium* sp. 1959, *Haemophilus parahaemolyticus*, *Klebsiella michiganensis*, *Moraxella bovis*, *Variovorax* sp. SRS16
*aph(3’)-Ia*	*Corynebacterium* sp. 1959, *E. coli*, *Klebsiella quasipneumoniae*, *Variovorax* sp. PAMC28562
*aph(3’)-IIa*	*E. coli*
*aph(3’)-IIIa*	*E. faecium*, *S. aureus*, *Streptococcus agalactiae*
*aph(6)-Id*	*Corynebacterium* sp. 1959, *K. michiganensis*, *Neisseria shayeganii*, *Providencia rettgeri*, *Variovorax* sp. SRS16
*blaOXA-2*	*A. baumannii*, *A. hydrophila*, *P. aeruginosa*
*blaOXA-85*	*Fusobacterium ulcerans*
*blaOXA-119*	*Geobacter sulfurreducens*
*blaOXA-347*	*Alistipes shahii*, *B. fragilis*, *Bacteroides heparinolyticus*, *Capnocytophaga* sp. H2931, *Capnocytophaga* sp. H4358, *C. stomatis*, *Chryseobacterium* sp. POL2, *Elizabethkingia anophelis*, *Empedobacter brevis*, *Myroides odoratimimus*, *R. anatipestifer*
*blaROB-1*	*Actinobacillus pleuropneumoniae*, *Conchiformibius steedae*, *Glaesserella parasuis*, *Haemophilus haemolyticus*
*blaROB-9*	*G. parasuis*
*blaROB-10*	*Bibersteinia trehalosi*
*blaTEM-116*	*E. coli*
*catIII*	*K. michiganensis*
*cfxA2*	*Capnocytophaga cynodegmi*, *Parabacteroides distasonis*, *Porphyromonas cangingivalis*, *Porphyromonas crevioricanis*, *Porphyromonas gingivalis*, *Tannerella forsythia*
*dfrA14*	*K. michiganensis*, *Ochrobactrum anthropi*
*emrE*	*E. coli*
*emrK*	*E. coli*
*ereA*	*Geobacter daltonii*
*ermB*	*Enterococcus gilvus*, *Enterococcus* sp. FDAARGOS_375, *S. agalactiae*, *Streptococcus suis*
*ermF*	*A. shahii*, *B. fragilis*, *C. stomatis*, *C. indologenes*, *P. distasonis*, *P. cangingivalis*, *Prevotella intermedia*, *R. anatipestifer*
*ermG*	*C. difficile*
*ermX*	*Trueperella pyogenes*
*gadW*	*E. coli*
*gadX*	*E. coli*
*lnuB*	*S. suis*
*lnuC*	*Streptococcus equi*, *Streptococcus gwangjuense*
*lsaE*	*S. suis*
*mdtN*	*E. coli*
*mef(En2)*	*B. fragilis*, *P. cangingivalis*, *P. gingivalis*, *P. intermedia*
*mel*	*Streptococcus pluranimalium*
*pgpB*	*P. gingivalis*
*qacL*	*P. aeruginosa*
*SAT-4*	*S. aureus*
*sul1*	*A. hydrophila*, *P. aeruginosa*
*sul2*	*Corynebacterium* sp. 1959, *H. parahaemolyticus*, *K. michiganensis*, *M. bovis*, *Pasteurella multocida*, *P. rettgeri*
*tet32*	*Blautia hansenii*, *Bulleidia* sp. zg-1006, *C. difficile*, *Clostridium cellulovorans*, *Eubacterium maltosivorans*, *Eubacterium* sp. NSJ-61, *Faecalibacterium prausnitzii*, *Lachnoanaerobaculum umeaense*, *Peptoclostridium acidaminophilum*, *Roseburia intestinalis*, *Streptococcus anginosus*, *Streptococcus constellatus*, *S. equi*
*tet44*	*A. intestinale*
*tetH*	*Proteus vulgaris*, *Pseudomonas putida*
*tetM*	*C. difficile*, *Enterococcus faecalis*, *Mogibacterium pumilum*, *Streptococcus* sp. FDAARGOS_521
*tetO*	*C. difficile*, *Enterococcus hirae*, *Murdochiella vaginalis*, *Streptococcus acidominimus*, *S. anginosus*, *S. constellatus*, *S. equi*, *S. suis*
*tetQ*	*Alistipes indistinctus*, *Bacteroides dorei*, *B. heparinolyticus*, *Bacteroides ovatus*, *Bacteroides* sp. HF-5287, *Phocaeicola coprophilus*, *P. crevioricanis*, *Prevotella fusca*, *P. intermedia*
*tetS*	*Streptococcus parauberis*
*tetW*	*Enterocloster bolteae*, *F. prausnitzii*, *Megasphaera stantonii*, *S. suis*
*tetWNW*	*Filifactor alocis*, *M. pumilum*
*tetX*	*B. fragilis*, *P. distasonis*, *P. intermedia*, *R. anatipestifer*
*tetX4*	*R. anatipestifer*
*tetX5*	*B. fragilis*, *C. stomatis*, *R. anatipestifer*
*tetZ*	*Rothia nasimurium*
*ugd*	*E. coli*

## Data Availability

The short-read data of the sample data are publicly available and can be accessed through the PRJNA648123 [24] and PRJNA683923 [25] from the NCBI Sequence Read Archive (SRA).
